# A perspective: Challenges and opportunities of a novel national dental benefit

**DOI:** 10.3389/froh.2023.1207581

**Published:** 2023-05-17

**Authors:** Anna Ness, Kamila Sihuay-Torres, Sonica Singhal

**Affiliations:** ^1^ Faculty of Dentistry, University of Toronto, Toronto, ON, Canada; ^2^ Public Health Ontario, Toronto, ON, Canada

**Keywords:** public health dentistry, healthcare policy, oral health, dental care, dental program, health equity, Canada

## Abstract

In Canada, the federal government launched the interim Canada Dental Benefit (CDB) on December 1, 2022, to support access to dental care for children <12 years. The interim benefit shows government's assurance to develop a long-term national dental care program. The benefit will be a cash transfer through Canada's revenue services agency, ranging from $260 to $650 annually. This perspective examines the federal initiative and reflects on its strengths and challenges to learn lessons, which can support the long-term solution that is being currently planned. This article outlines a number of positive aspects as well as challenges from the perspectives of varied stakeholders; the feasibility of the application process; remaining potential gaps due to restricted eligibility criteria; possible effects of unrestricted oral health care services and reimbursement rates; valuing of patient autonomy; guidelines for the expansion of the program to other populations; and remaining barriers to oral health care access are analyzed. The CDB is cause for excitement for the Canadian population because it is an opportunity to reduce affordability barriers to accessing dental care. That said, it is important to discuss anticipated challenges and indirect consequences, particularly through the lens of equity, to support the new CDB and the proposed national dental care program in achieving the much-awaited goal of putting the mouth back into the body.

## Introduction

On March 22, 2022, for the first time in Canada's history, oral health gained attention at the federal level (1). To be precise, a commitment of $5.3 billion was made “to provide dental care for Canadians with family incomes of less than $90,000 annually, starting with those under 12 years old in 2022, expanding to under 18 years old, seniors and persons living with a disability in 2023, with full implementation by 2025.”(2) Following that, consultations with relevant stakeholders including provincial/territorial governments, dental associations, and insurance companies were arranged to assess how this program will roll out (3). However, as the time was ticking fast to fulfill the commitment to start the program for children under 12 years by December 2022, the government announced the Canada Dental Benefit (CDB), an interim plan, on September 13, 2022 (3).

Beginning December 1, 2022, middle and low-income families earning less than $90,000 were eligible to receive the CDB for every child twelve years of age or under. The CDB is a direct payment, tax-free credit ranging from $260 to $650 per child, depending on the household income bracket. Approximately 500,000 Canadian children would benefit from this targeted investment of $938 million. Families are able to receive the benefit retroactively for completed dental services dating back to October 1, 2022 (3–5).

To be clear, the CDB is not the first phase of the proposed Canadian Dental Care Program, but the interim stopgap plan (3). This credit is publicly financed and administered through the federal government, but delivery of dental treatment will remain in private dental offices situated in provinces and territories (3). Meaning that this benefit can be requested before or after accessing dental services (3–5). This is a huge endeavour at the federal level and a remarkable first in the Canadian history. However, it is necessary to review it more keenly to assess potential challenges and indirect consequences of this initiative, which in turn would support in assessing the opportunities for the longer term definitive national dental care program, which is expected to put the mouth back into the body by being effective, efficient, and sustainable.

## Anticipated challenges, indirect consequences, and potential opportunities

### Online application through Canada Revenue Agency platform

Accessing the Canada Revenue Agency account online will likely be the fastest and most efficient way of applying for the benefit; however, this tax assessment eligibility will automatically put new immigrant families in the disadvantaged situation. Getting a support like the CDB for young children can be crucial for newcomer families; depriving them of this benefit can create inequities. Those who have dependents nearly approaching their 12th birthday would be particularly disadvantaged, as they would not be able to receive this benefit. A potential solution can be that those families who had any expenses for eligible children from October 1, 2022, to the time they have filed their 2022 taxes, can claim those expenses later. Another option can be, that along with all the other requirements, they can show the proof of becoming permanent residents in the year 2022 instead of a tax assessment of 2021, to make this benefit equitable for them.

In addition, there might be people who did not qualify for this benefit based on their 2021 income; however, their financial circumstances might have changed in 2022, putting them at need of receiving this need. A provision of eligibility based on current income status of family would have been more inclusive, especially in the times when COVID-19 had brought unprecedented circumstances to many families.

### Benefits are based on the family income and not on the clinical need

As we described above, this model is based on family income and the benefit could vary from $260 to $650 per child depending on the income bracket. Within one benefit bracket, children who need more urgent treatment will receive the same amount of money as children who do not. Even though parents could ask for more money in the second benefit period, the severity of the disease could get worse if they must wait until they receive more money. Children who will turn 12 by the second benefit period would be especially disadvantaged as they will lose their eligibility without getting the treatment complete. For the same reason, some Canadian jurisdictions pay a set amount every two years rather than annually (6, 7). On similar lines, a potential solution could be that those children whose dental needs are not met with the amount allocated in the first year could ask for advance payment for the second benefit period to get the treatment completed in time. As such, the amount received in the first benefit period may not be enough especially for those families, who are eligible for only $260 per child, because the complete examination and radiographs may itself result in more amount than that.

### Open ended dental service coverage

There are currently no limitations on what type of dental services would be covered through the CDB, which is understandable as this is not a public dental program which have a defined schedule and are generally restrictive in the services coverage. Rather, this benefit will put money directly into people's pocket, who would be able to get their children needed dental care, without compromising their other daily essential expenses. That said, setting no limitations or criteria on what services would be eligible to be paid through this benefit has potential to cause some harm to the targeted population, especially because this benefit will be replaced by a definitive program, which like other public programs, may cover restricted services. For example, orthodontic treatment in general is quite expensive and is not covered through most public programs. According to the Canadian Health Measures Survey Cycle 1, among 6–11 years old, 10% children from high income families and 6% from middle income families reported receiving orthodontic treatment; however, the numbers beings very small were not reported for low-income families, which reflects their unaffordability for such treatment (8). With this benefit, low-income families may get the opportunity to get their children's teeth fixed. As orthodontic treatment is generally installment based, they may plan that initial payments can be made through this interim benefit and the rest would be paid through the definitive program, which would be launched by July 2024, with this assumption that the definitive program will have same services coverage as the current benefit. If the proposed program does not include orthodontic treatment, a number of children may not be able to complete their treatment, making them further disadvantaged. Therefore, it is very important to ascertain the service basket for the definitive program much in advance and to align the CDB with it.

### Differences between tax benefits and publicly financed dental programs

The CBD is a credit earmarked to be used specifically for dental care services, making it different than other tax-credits which are not given for any specific usage. For example, the Canada Child Benefit is transferred to families in the same manner as the CDB but holds no conditions by which the money is to be spent (9). This is understandable; however, as oral health is crucially dependent on daily oral hygiene practices, which needs resources such as toothpaste, toothbrush, dental floss, and mouth wash, it would be beneficial, if the CBD credit can also be utilized for purchasing such products facilitating families to enhance their oral hygiene practices.

Similarly, the CDB is markedly different from publicly financed dental care programs. While both have eligibility criteria regarding mean family income, the criteria for the CDB are much less financially stringent. For example, to be eligible for the Health Smiles Ontario, a publicly funded dental program in Ontario, Canada, a family with two children must show a yearly family net income of $26,817 or less (10), which is strikingly lower than the family income criteria in the CDB. In addition, publicly financed dental programs are restricted in the basket of services covered, as previously discussed. In sum, the inclusion of a larger family income and the lack of rigidity of the CBD ensures that more Canadians have access to dental treatment than ever before.

Lastly, according to the proposed bill, families who will not use the whole benefit do not have to repay the excess. When resources are finite or rather limited, gaining allocative efficiencies is crucial to ensure the sustainability of any intervention. For that reason, in the future, as definitive dental program is launched, it must establish a dental services schedule that is scientifically and ethically defensible (11). This will support the publicly funded dental care program to be utilized more equitably and efficiently. However, to accomplish this, professional expertise (through extensive consultations with dental associations and surveys among dental professionals) and public opinion (through surveys and focus groups discussions) would be required. If tax payers' dollars are being invested to fund the program then it is important to understand what dental care services do tax payers' value the most. Also, dental professionals being the experts in the field and knowing the normative needs of their population should be consulted to understand what services be essentially covered in the definitive program.

### Reimbursement rates

The interim CDB has no mention of the reimbursement rates, which is again understandable as this benefit is a direct cash transfer to eligible people and not exactly a dental care coverage program like any other public program, where reimbursement rates are decided. That said, it would be difficult for dental care providers to decide how to charge those patients who are enrolled in some other public program and qualify for this benefit too; should they charge as per the public program fee guide or the regular one. How about the remaining care, if they do not accept other public programs but are ready to provide services which can be covered through this benefit? Some advanced thinking is needed to make these provisions more seamless.

### Public program coverage does not affect eligibility, but private coverage does

As per the eligibility criteria, children who have coverage under any public program remain eligible for this program but having any benefit through private insurance (including employer coverage) makes them ineligible. This can have some unintended inequitable consequences. Children whose families' annual income is less than 70,000, but not eligible for a public program, would have their affordability limited to services worth $650 vs. children enrolled in any public program would be able to afford much more as a number of services would be covered by program and for the uncovered they can pay out of pocket through this benefit. As such, with most low-income jobs, where employer covers dental benefits, the co-pay at the point of delivery is substantial (20%–50%) to deter someone to utilize that benefit.(12) If families eligible for this benefit could pay those co-payments from this CDB, could have been a big support in accessing dental care. Also, it may prompt people either not to disclose their private insurance or tend to opt out of employer-based insurance coverage, which generally involves a monthly premium co-paid by employee and employer. Insurance companies, to compensate for the lost business, may increase insurance premiums for middle- and high-income employees, thereby making dental care less affordable for them.

### Accounting of the CDB

The Canadian dental care system is mostly privately administered, funded, and delivered. To be precise, 94% of dental care is privately financed and 6% from the public purse. Within that 94%, 60% of funds are paid through employer-based insurance and 40% is paid out-of-pocket (13). With the introduction of this CDB, the publicly funded proportion for dental care expenses is expected to increase; however, would not be reflected so, as this money will be a direct fund transfer to parents/guardians of eligible children. Though money would come from a public account, parent/guardians when paying out-of-pocket at a fee-for-service dental practice, will be accounted accordingly. The government though can show through the Canada Revenue Agency account how much funding was transferred by the end of benefit period; however, looking at the financial books of dental care delivery, ascertaining the proportion of out-of-pocket funding actually coming from the government would be difficult.

### Children under 18, seniors, and people living with disabilities

As mentioned above, the commitment made on March 22, 2022, stated that the dental care program would start with children under 12-year-olds in 2022, expanding to under 18-year-olds, seniors and persons living with a disability in 2023. However, the interim benefit, whose enrollment started on December 1, 2022, would be only for children under 12 years. This interim benefit has been proposed for up to June 30, 2024, but there is no mention if in the second benefit period, i.e., July 1, 2023, to June 30, 2024, it would expand to children under 18, seniors, and people living with disabilities, as had been originally committed. If the benefit is not extended to other proposed populations in the second benefit period, it may be challenging for those vulnerable populations, who have been waiting for long to access unaffordable dental care.

### Empowering patient autonomy

In contrast to public programs offered by provinces or territories, this benefit will encourage the autonomy of the patients by providing them with a direct cash transfer to receive dental care. Patient autonomy is described as the capacity to act, which may be expressed in different ways, for instance taking “initiative, responsible agen[cy], participation and taking an active role in a decision concerning their care or shaping their own life” (14). Empowering patient autonomy helps them to value their own decision making as it pertains to oral health care, which is a positive departure from the traditional model of care in which the dentist holds professional dominance over patient decision making which does not always promote agency or empowerment on behalf of the patient. This diversion from the routine though could be greatly appreciated, could create more distress when the definitive program would be launched which probably would be restrictive in its coverage of services and the financial transaction would be between the provider and the administrator, making eligible clients lose their autonomy and be dependent on the insurer and provider to determine their dental treatment needs vs. choices.

### Barriers to dental care access

Access is influenced by a certain set of factors that determine both one's ability to enter and use of the health care system. These factors are: accessibility, affordability, availability, accommodation, acceptability and awareness (15, 16). If access is equitable, meaning equal and fair, motivation alone pushes someone to use health services. Using this model to analyze the CDB, we can assess the benefit as a policy endeavor aimed to combatting financial barriers (i.e., affordability) but limited in ameliorating other barriers. Again, this is because the CDB is not a health program or an insurance plan, but rather a modest boost of cash. To be sure, financial credit is a constructive method to improve access to care that has been advocated for because it removes price barriers at the point-of-care (17). However, this could lead to unintended results. For example, low-income families living in rural or remote areas may be eligible for the CDB but may not be able to afford the current price of gas to travel to their dental office and use the benefit for dental treatment (that is, a restriction to accessibility). Similarly, if families are not made aware of this credit, they won't apply for it (that is, a restriction to awareness).

Along those same lines, families who prefer seeking dental treatment in public clinics would be hindered from using funds from the CDB in this setting because public dental clinics in Canada do not accept out-of-pocket payments. An unintended consequence of this may exclude people from seeking treatment if they typically see a dentist in a public setting (that is, a restriction to acceptability). For example, a study found that socially vulnerable people, particularly from low-income families, showed some preference for seeking treatment in a public clinic (18). This leads us to believe that future iterations of the CDB or the National Dental Care Program should be permissive in their capacity to validate patient preferences. Provisions can be made to allow public clinics to also utilize a fee-for-service model along with salaried/contracted model to accommodate all Canadians irrespective of their insurance status. This can be an opportunity for a public—private partnership, which will provide true autonomy to Canadians in selecting their provider. What is more, accepting out-of-pocket payments increases the resources of the clinic which will help to provide care to more people in need of dental services not currently part of the public system and will generally promote financial liquidity—monies which can be utilized by the clinics to use how they see fit.

## Conclusions

The CDB is cause for excitement for the Canadian population because it is an opportunity to reduce affordability barriers to accessing dental care. To recap, it is necessary to highlight that this benefit differs from other social assistance programs because it is cash-transfer directly to the patients. In that sense, the CDB encourages the patient's autonomy to let them make their own decisions regarding their oral health care. However, moving on to the following stages of the benefit requires increasing eligibility for other vulnerable populations.

Going forward, we see two pathways toward the National Dental Care program emerging from the interim CDB. In the first pathway, the National Dental Care Program would be modeled on other social assistance programs. This pathway would have more challenges in its wake, such as decision-making regarding guidelines on dental service coverage and reimbursement rates. Conversely, the second pathway would see the progressive inclusion of the rest of the population *via* a direct cash benefit to the patients, as with the interim program. In that way, the interim program would become the definite program. Perhaps, this second pathway would meet less resistance toward effective implementation, which would be a quicker and more effective way to deliver a dental benefit in a way that would permit equitable access to dental care to more Canadians than ever before.

## Data Availability

The original contributions presented in the study are included in the article, further inquiries can be directed to the corresponding author.
